# Learning to control ICP

**DOI:** 10.1186/2045-8118-12-S1-P12

**Published:** 2015-09-18

**Authors:** Jinendra Ekanayake, Aswin Chari, Claudia Craven, Simon D Thompson, Syed N Shah, Neekhil A Patel, Samir A Matloob, Huan-Wee Chan, Edward W Dyson, Ahmed K Toma, Laurence Watkins

**Affiliations:** 1 National Hospital for Neurology and Neurosurgery, UK

## Introduction

The landmark discovery that control of autonomic physiology could be ‘learned’ using biofeedback was first demonstrated with heart rate [1,2]. Biofeedback control has since been demonstrated with physiological variables such as regional cerebral blood flow, and end tidal carbon dioxide, with therapeutic application in conditions including migraine and epilepsy [3-6]. Here, we demonstrate for the first time, learned control of intracranial pressure (ICP), in a single patient using biofeedback of simultaneous ICP recordings via a Speigelberg™ intracranial pressure monitor.

## Hypothesis

Mindfulness of breathing, guided by analogue ICP biofeedback can be used to reduce ICP in patients with known idiopathic intracranial hypertension.

## Method

A single patient, with a known history of idiopathic intracranial hypertension was trained to direct attention to their breathing, while simultaneously observing their ICP, as displayed on a analogue readout. The display was connected to a right frontal Spielberg intracranial pressure monitor. In addition to an explicit instruction to direct their attention to the passage of breath through their nostrils, and maintaining regular breathing, the patient was asked to reduce their ICP value as much as possible, including negative values. This was repeated over two days. Specifically they performed 3 sessions on the 1st day, and 2 sessions on the 2nd day – each session consisted of 5 blocks of 90s each of attempted ICP reduction using ‘biofeedback-guided mindfulness’, followed by 60s of rest.

## Results

During ‘biofeedback-guided mindfulness’, the patient reduced their median ICP on both days i.e. session averages: Day 1 Median ICP 1.9, Day 2 Median ICP -2.63. Control values were obtained from the hour before and after the biofeedback sessions i.e. average: Day 1 Pre-biofeedback Median ICP 6.5, Post-biofeedback Median ICP 3.5, Day 2 Pre-biofeedback Median ICP 5.0 Post-biofeedback Median ICP 4.1 (Overall day averages, Day 1 Median ICP 5.9, Day 2 Median ICP 6.0).

## Conclusion

Using mindfulness of breathing and biofeedback of simultaneous ICP recordings, we were able to train a patient to control their own ICP. Although this will require validation with more patients, it provides for the possibility of training volitional control and reduction of symptomatic increases in ICP.
